# Genetic Polymorphisms of *MMP1*, *MMP9*, *COL1A1*, and *COL1A2* in Polish Patients with Thoracic Aortopathy

**DOI:** 10.1155/2020/9567239

**Published:** 2020-09-24

**Authors:** Iwona Gorący, Seweryn Grudniewicz, Krzysztof Safranow, Andrzej Ciechanowicz, Paweł Jakubiszyn, Anna Gorący, Mirosław Brykczyński

**Affiliations:** ^1^Department of Clinical and Molecular Biochemistry, Pomeranian Medical University, Szczecin, Poland; ^2^Department of Cardiac Surgery, Pomeranian Medical University, Szczecin, Poland; ^3^Department of Biochemistry and Medical Chemistry, Pomeranian Medical University, Szczecin, Poland

## Abstract

**Background:**

The pathogenesis of thoracic aortopathy is complex, and much evidence suggests the influence of genetic factors. Some genes with polymorphisms are widely considered critical factors in the initiation and development of aortic aneurysm. The aim of our study was to analyze the association of genetic polymorphisms of *MMP1* rs1799750 (c.-1607G>GG), *MMP9* rs3918242 (c.-1562C>T), *COL1A1* rs1800012 (c.1245G>T), and *COL1A2* rs42524 (c.1645G>C) with predisposition to thoracic aortopathy in Polish patients and with clinical characteristics of these patients.

**Methods:**

The study was carried out with 96 patients with thoracic aortopathy (47 patients with ascending aortic aneurysm and 49 patients with thoracic aortic dissection) and 61 control subjects without thoracic aortopathy. The *MMP1*, *MMP9*, *COL1A1*, and *COL1A2* polymorphisms were determined by PCR-RFLP.

**Results:**

No significant differences in the frequency distributions of *MMP1*, *MMP9*, *COL1A1*, and *COL1A2* genotypes or alleles were found (1) between the control group and patients with ascending aortic aneurysm (AsAA), (2) between the control group and patients with thoracic aortic dissection (TAD), or (3) between AsAA and TAD patients. Multivariate logistic regression analysis revealed that *MMP1* and *MMP9* polymorphisms were associated with the degree of aortic valve regurgitation.

**Conclusion:**

The results of our study did not support associations between *MMP1*, *MMP9*, *COL1A1*, and *COL1A2* genetic variants with the risk of thoracic artery disease in Polish patients. However, rs1799750 *MMP1* and rs3918242 *MMP9* seem to be associated with the degree of aortic regurgitation.

## 1. Introduction

Thoracic aortic disease (or thoracic aortopathy) is a collective name which includes aortic aneurysm and acute thoracic aortic dissection (TAD) [1]. The ascending aorta is the segment most commonly affected by thoracic aortopathy. It is known that environmental factors contribute to the formation of aneurysms and aortic dissection including age, smoking, hypertension, trauma associated with high overload and vessel inflammation, obesity, familial history, atherosclerotic cardiovascular disease, and family history [2]. However, knowledge concerning these risk factors is not yet complete.

The pathomechanisms of the formation of aortic aneurysms involve a weakening of the aortic wall as a result of reduction in the amount of elastic fibers and smooth muscle, the effect of tensile stresses, and the accumulation of proteoglycans [3]. Proteolytic degradation of the aortic wall plays a crucial role in the pathogenesis of thoracic aortopathy. Matrix metalloproteinases (MMPs), the zinc-dependent proteases, are important enzymes that control the remodeling or degradation of the extracellular matrix (ECM). The MMPs demonstrate a critical role in remodeling of the ECM by proteolytic degradation of its components including collagen and elastin. They regulate activity of other proteases, growth factors, chemokines, and cell receptors [4]. High activity of these enzymes occurs in various pathological conditions, especially in myocardial injury, tumor metastases, aneurysms, and inflammation [5, 6]. Zhang et al. described the functional polymorphism of *MMP9*: c.-1562C>T (rs3918242) demonstrating that the T allele gives a higher enzyme activity [7]. Additionally, the functional polymorphism *MMP1*: c.-1607G>GG (rs1799750) is associated with increased *MMP1* transcription [8]. Karapanagiotidis et al. [9] reported that levels of MMP1 were found to be lower in healthy individuals compared to patients with acute and chronic aortic dissection, aortic aneurysm, and myocardial ischemia. In addition, the *MMP9* genetic polymorphism has been correlated with thoracic aortic disease [10]. Therefore, MMPs, especially MMP1 and MMP9, have been widely considered critical factors in the initiation and development of aortic aneurysms [9, 10].

Collagen type I (COL1) has long been suggested to be involved in aneurysm pathogenesis. Type I collagen provides the main protein structure of connective tissue, and it is the most abundant fibrillar collagen in vertebrates, encoded by two genes, the *COL1A1* and the *COL1A2* genes that express the *α*1(I) and *α*2(I) chain, respectively. It has been shown that polymorphisms of these type I collagen genes are associated with vascular diseases [11]. A recent meta-analysis suggests that *COL1A2* rs42524 is a significant risk factor for intracranial aneurysm susceptibility, with an especially strong effect in Asian people [12]. Lindahl et al. showed that genetic variants of *COL1A2* gave an increased risk of stroke, myocardial infarction, and lower bone density [13]. The type I collagen-related diseases are characterized by a wide spectrum of diseases and high clinical variability, whose genetic basis is still poorly understood. Several candidate genes have been identified as being associated with thoracic aortic aneurysms and bicuspid aortic valve (BAV) [14, 15].

It should also be highlighted that thoracic aneurysms are more common in individuals with BAV. BAV is a common congenital cardiovascular occurrence found in 1-2% of the general population [16]. On the other hand, the incidence of aortic dissection in BAV patients is low but higher than that in the general population [17].

Aortic lumen diameter is a modestly effective marker of aortic risk but must be used in combination with other potential risk factors (e.g., presence of coarctation, family history of dissection, or aortic root phenotype) for individualised risk [18]. It is believed that characterization of novel aortic risk markers (e.g., laboratory biomarkers, genetic biomarkers, and wall stress characterization) will be central to the management of these patients.

Therefore, the aim of our study was to analyze the association of genetic polymorphisms of *MMP1* rs1799750 (c.-1607G>GG), *MMP9* rs3918242 (c.-1562C>T), *COL1A1* rs1800012 (c.1245G>T), and *COL1A2* rs42524 (c.1645G>C) with predisposition to thoracic aortopathy and with the clinical characteristics of these patients.

## 2. Materials and Methods

### 2.1. Patients

The study was conducted in accordance with the Declaration of Helsinki and was approved by the bioethics committee at the Pomeranian Medical University in Szczecin. Informed consent was obtained for patients and for control subjects. This was a prospective study of 96 consecutive patients undergoing surgery for thoracic aortopathy in the Department of Cardiac Surgery of the Pomeranian Medical University. The study group (64 men and 32 women aged 36-72 years) consisted of 47 patients with ascending aortic aneurysms (AsAA) and 49 patients with thoracic aortic dissection (TAD). Individual indications for surgery were determined on the basis of body surface area (BSA) measurements and the size (width sinotubular junction) of the relevant parts of the aorta [19]. For simplicity, the norm was calculated as 19 mm/(1 m^2^ BSA) for the aorta and 15 mm/(1 m^2^ BSA) for the ascending aorta. Aortic dilatation was diagnosed when width exceeded 100%; a result more than 150% of this norm was defined as aneurysm. In accordance with the guidelines, we used computed tomography for imaging the aorta and for assessing the aortic echocardiography (including AsAA and TAD) [20]. Familial, inflammatory, and traumatic AsAA were excluded from the study.

All patients had a complete 2-dimensional M-mode echocardiography performed (using an Acuson Sequoia 512 unit; Siemens, Munich, Germany; equipped with a 2-4 MHz imaging transducer). Measurement techniques were consistent with American Society of Echocardiography conventions [21]; in unclear cases, computed tomography was performed. The control group consisted of 61 patients (26 men and 35 women aged 36 to 72 years) with nonspecific chest pain in whom ischemic heart disease was excluded by coronary angiography. In addition, the control subjects underwent echocardiography to exclude bicuspid aortic valve and other heart valve malformations.

Full medical history was collated, including arterial hypertension defined as systolic blood pressure greater than 140 mmHg and/or diastolic blood pressure greater than 90 mmHg, or a reported history showing hypertension. Body mass index (BMI) was calculated as (mass, kg)/(height, cm)^2^. Patients were classified as “current smokers” if they reported a daily rate of more than five cigarettes. The summary of clinical characteristics of AsAA patients, TAD patients, and controls is provided in Table 1.

### 2.2. Genotyping

Genomic DNA was isolated from venous peripheral blood leukocytes (using a QIAamp DNA Mini Kit; Qiagen, Hilden, Germany). The polymorphisms *COL1A1* rs1800012 (c.1245G>T), *COL1A2* rs42524 (c.1645G>C), *MMP1* rs1799750 (c.-1607G>GG), and *MMP9* rs3918242 (c.-1562C>T) were analyzed by PCR and restriction fragment length polymorphism (RFLP) analysis (all rs numbers are from the dbSNP database, (http://www.ncbi.nlm. http://nih.gov/snp). The primer pairs (TIB MOL BIOL, Poznań, Poland) used were for *COL1A1* rs1800012: forward 5′-GGAAGACCCGGGTTATTTGC-3′ and reverse 5′-CGCTGAAGCCAAGTGAAATA-3′; for *COL1A2* rs42524: forward 5′-AGTAATACCTGAGGCTTTGAGACA-3′ and reverse 5′-GAGAGGTACGGTATGGTGATTTA-3′; for *MMP1* rs1799750: forward 5′-GAAATT GTAGTTAAATCCTTAGAAAG-3′ and reverse 5′-TATGGATTGCTGTTTTCTTGC-3′; and for *MMP9* rs3918242: forward 5′-GCCTGGCACATAGTAGGCCC-3′ and reverse 5′-CTTCCTAGCCAGCCGGCATC-3′. The PCR products were digested with restriction enzymes Van91I, BseDI, EcoNI, and HaeIII, respectively (MBI Fermentas, Vilnius, Lithuania), and the digestion products were separated in 3% agarose gels. Both negative (no DNA template) and positive (genotype-confirmed DNA template) control samples were used in the PCR-RFLP analyses. All samples were independently genotyped using a blind method in duplicate.

### 2.3. Statistical Analysis

Univariate analyses comparing groups were performed with Fisher's exact tests or chi-squared tests for qualitative variables and Mann-Whitney tests for quantitative variables. Genotype-phenotype associations were analyzed using dominant, recessive, and additive models. *p* < 0.05 was considered statistically significant without correction for multiple testing. Since four SNPs and two phenotypic features (bicuspid aortic valve and degree of aortic valve regurgitation) were analyzed, the Bonferroni-corrected significance threshold was 0.05/(4 × 2) = 0.006. Logistic regression was used for multivariate analysis with dichotomous dependent variables, and *p* values were calculated using the Wald tests.

## 3. Results

Characteristics of the studied patients are shown in Table 1. No significant differences in age, prevalence of hypertension, Ao max, or the frequency of large amounts of regurgitation from the aortic valve (AVR ≥ 2) were found between the control group and AsAA patients or between the control group and TAD patients. The percentage of males and smoking prevalence in the AsAA group was significantly higher compared to that in the control group. On the other hand, the frequency of diabetes mellitus in TAD patients was significantly lower compared to that in controls. No significant differences in analyzed variables were found between the AsAA and the TAD groups except for BMI, the frequency of diabetes mellitus, and BAV prevalence. The BMI values, the BAV frequency, and the diabetes prevalence in AsAA patients were significantly higher compared with those in the TAD group.

Characteristics of the patients with aortopathy in regard to type of aortic valve (BAV versus normal tricuspid aortic valve (TAV)) or to degree of aortic regurgitation (AVR ≥ 2 versus AVR ≤ 1) are shown in Table 2. No significant differences in the majority of analyzed variables were found between BAV subjects and TAV subjects. Only the frequency of diabetes mellitus, coronary artery disease, and the AsAA prevalence were significantly higher, and the prevalence of hypertension was significantly lower in BAV patients as compared to TAV patients. No significant differences in all analyzed variables were found between AVR ≤ 1 patients and AVR ≥ 2 patients.

The genotype distributions of *MMP1* rs1799750, *MMP9* rs3918242, *COL1A1* rs1800012, and *COL1A2* rs42524 in the studied group (controls+AsAA+TAD) conformed to expected Hardy-Weinberg equilibria (*p* = 0.530, 0.412, 0.890, and 0.304, respectively).

No significant differences in the frequency distributions of *MMP1*, *MMP9*, *COL1A1*, and *COL1A2* genotypes or alleles were found between the control group and AsAA patients, between the control group and TAD patients, or between the AsAA and TAD patients (Table 3). Therefore, further statistical analyses were carried out using a combined AsAA+TAD group consisting of 96 patients with thoracic aortopathy (47 AsAA patients and 49 TAD patients). No significant differences in the frequency distributions of *MMP1*, *MMP9*, and *COL1A1* genotypes or alleles were found between the BAV and TAV groups. However, significant differences were found in the frequency distributions of *COL1A2* genotypes and alleles between BAV and TAV patients using chi-squared test (genotypes) or Fisher's exact test (alleles) (Table 4). These differences did not remain significant after Bonferroni correction for multiple testing (*p* > 0.006). No significant differences in the frequency distributions of *MMP9* and *COL1A2* genotypes or alleles were found between the AVR ≥ 2 and the AVR ≤ 1 groups. However, significant differences were found in the frequency distributions of *MMP1* and *COL1A1* genotypes and *MMP1* and *COL1A1* alleles between AVR ≥ 2 and AVR ≤ 1 patients (Table 5). However, multivariate logistic regression analysis with Bonferroni correction for multiple testing (Table 6) revealed that only differences for *MMP1* and *MMP9* remained significant (*p* < 0.006).

## 4. Discussion

The key to the formation of an aortic aneurysm is a weakening of the aortic wall as a result of the reduction in the amount of elastic fibers and smooth muscle and the effects of tensile stresses leading to damage to the middle layer of the aorta [10]. The development and progression of thoracic aortopathy results from a combination of inflammation, hemodynamic stress, environmental risk factors, and genetic risk factors. In this study, we assessed the interaction between *MMP1*, *MMP9*, *COL1A1*, and *COL1A2* polymorphisms and the predisposition to thoracic aortopathy or with the clinical characteristics of these patients. We did not find a correlation between the studied genes and the predisposition to aortopathy. However, we have shown that the MMP1 and MMP9 genes may partially contribute to the incidence of aortic regurgitation.

Hereditary factors, e.g., bicuspid aortic valve or patent ductus arteriosus, also play an important role in the pathogenesis of thoracic aneurysms. Bicuspid aortic valve is the most common congenital heart defect [22, 23] and is considered heritable [16]. It is known to exhibit many phenotypic variations, which suggests a complex pathogenesis resulting at least partly from genetic variants. However, the real factors leading to impaired development towards a bicuspid aortic valve are not clear and most cases of BAV are sporadic. Earlier studies identified familial clustering of BAV and its occurrence in monozygotic twins, supporting an underlying genetic abnormality [24, 25]. It is also worth emphasizing that family members in whom BAV is inherited have a greater risk of aortic aneurysm despite an absence of a bifocal aortic valve [26]. The genetic components participating in the pathogenesis of this cardiac malformation have been identified by several studies including on *NOTCH1*, a gene encoding a signaling transmembrane receptor; *ACTA2*, a contractile protein; *TGFB2*, a signaling pathway protein; *FNB1*, a matrix protein; *KCNJ2*, a potassium channel; *GATA5*, a transcription factor; and *NKX2-5*, a transcription factor [14, 27]. Many studies have focused on changes in the signaling pathways in smooth muscle cells (SMCs); however, the importance of structural elements of cells and vascular tissues and their contribution to the initiation and progression of BAV have not been analyzed in detail.

Due to the wide distribution of type I collagen in the body, the effects of genetic variants that cause disturbed production and stability and hence disturbed function are not only limited to the vascular wall but theoretically should also affect other tissues. Its variants may also partially affect the development of valves and their proper functioning. It has been demonstrated that osteogenesis imperfecta in humans, which is characterized by a tendency for bone fractures, is caused by mutations in either the *COL1A1* or *COL1A2* genes encoding type I collagen [15]. It should also be noted that these patients sometimes develop aortic dissection [28] and, in addition, cases of the Ehlers-Danlos heart valve syndrome associated with genetic variants in various *COL1A1*-related genes have been described [29]. Many studies have shown that genetic variation in genes associated with extracellular matrix degradation, including MMPs, may contribute to different levels of expression in the aortic wall and the development of aneurysm. Some studies have indicated an increase in the concentrations of MMP1 and MMP9 in the walls of diseased aortas and in plasma in patients with acute thoracic aortic diseases [9, 30, 31]. However, other studies do not support this relationship. In our study, we did not detect a direct relationship between polymorphisms of *MMP1*, *MMP9*, *COL1A1*, and *COL1A2* and the occurrence of thoracic aortopathy.

This study has some limitations. First, the number of tested patients is relatively small. Also, it should be noted that environmental risk factors are widespread and strongly exhibited in Polish patients, and therefore, the impact of genetic components may be less pronounced. It should also be noted that the study group is heterogeneous in terms of the disease studied (ascending aortic aneurysm and aortic dissection), which may affect the results obtained.

However, our result is in harmony with other studies. Wang et al. [32] did not find any significant differences in genotype or allele frequencies of *MMP9* between TAD cases and controls; however, their results provided evidence for an association between *MMP9* rs2274756 and female TAD risk in the Chinese Han population. Additionally, no relationship was found via meta-analysis of *MMP1* and *MMP9* polymorphism studies with thoracic aortic aneurysmal diseases [33] and in a small preliminary study, researchers found no relationship between MMP1 and MMP9 levels and the elastic modulus of the aortic wall in patients with thoracic aortic aneurysm [34]. However, there are some studies in the literature that have confirmed a relationship between *MMPs* and the occurrence of thoracic aortic aneurysm [10, 35].

There are several possible explanations for the discrepancies listed above. For example, the associations between tested SNPs that are involved in tissue remodeling-related diseases might be ethnic-specific. Alternatively, the different results may result from different sample sizes, different study designs, gender differences, and exposure to risk factors of varying severity or simply random error. Favé et al. [36] studied a founder population in Quebec and demonstrated how the local environment directly influences disease risk phenotypes and that genetic variability, including less common variants, can modulate individual responses to environmental challenges. Other researchers have also shown that environmental factors not only directly affect phenotypic variation but can also modulate the links between segregating genetic variants and the phenotypes [37, 38].

Although in our study we did not show an effect of metalloproteinases in the development of aortic aneurysm, we have found associations between *MMP1* and *MMP9* polymorphisms with degree of aortic valve regurgitation. The conclusions to be drawn from this are not clear. This may be related to our relatively small study group in which we recorded a more frequent occurrence of BAV regurgitation. Our observation needs to be followed-up with a much larger sample size to establish an association.

## 5. Conclusion

The results of our study did not confirm associations between *MMP1*, *MMP9*, *COL1A1*, and *COL1A2* genetic variants with the risk of thoracic artery disease in Polish patients. However, *MMP1* rs1799750 and *MMP9* rs3918242 seem to be associated with the degree of aortic regurgitation in these patients.

## Figures and Tables

**Table 1:** Clinical characteristics of the studied patients.

Variable^∗^	Control group (*n* = 61)	AsAA (*n* = 47)	TAD (*n* = 49)
Age (years)	60 (36-78)	60 (28-79)	57 (31-81)
BMI (kg/m^2^)	27.3 (19.0-40.0)	28.3 (20.0-38.1)	26.1 (16.1-39.9)^a^
Males	26 (43%)	34 (72%)^c^	30 (61%)
Smoking	10 (16%)	17 (36%)^d^	15 (31%)
Diabetes mellitus	12 (20%)	7 (15%)	0 (0%)^a,c^
Hypertension	33 (54%)	34 (72%)	27 (55%)
BAV	-	24 (51%)	6 (12%)^a^
AVR ≥ 2^#^	-	35 (74%)	32 (65%)

AsAA: ascending aortic aneurysm; TAD: thoracic aortic dissection; BMI: body mass index; BAV: bicuspid aortic valve; AVR: aortic valve regurgitation. ^∗^Chi2 test for qualitative variables and Mann-Whitney test for quantitative variables. ^#^Aortic valve regurgitation (AVR) was measured using rank scale (“-” = 0, “+” = 1, “++” = 2, “+++” = 3, “++++” = 4), and the study group was divided, depending on the degree of regurgitation, into two subgroups: low regurgitation (AVR ≤ 1) and large (AVR ≥ 2) regurgitation. ^a^*p* < 0.01 as compared to the AsAA group; ^c^*p* < 0.01 and ^d^*p* < 0.05 as compared to the control group.

**Table 2:** Clinical characteristics of the patients with aortopathy in regard to the type of aortic valve or to the degree of aortic valve regurgitation.

Variable^∗^	TAV (*n* = 66)	BAV (*n* = 30)	*p*_B_	AVR ≤ 1 (*n* = 29)	AVR ≥ 2 (*n* = 67)	*p*_AI_
Age (years)	59 (28-81)	58 (33-78)	0.363	59 (37-81)	58 (28-79)	0.529
BMI (kg/m^2^)	26.8 (16.1-39.9)	28.1 (18.6-38.1)	0.226	27.4 (16.4-35.7)	26.8 (19.1-39.9)	0.395
Males	41	23	0.161	16/13	48/19	0.116
Smoking	22	10	1.000	8/21	24/43	0.432
Diabetes mellitus	2	5	0.018	4/25	3/64	0.107
Hypertension	47	14	0.021	16/13	45/22	0.262
CAD	8	10	0.014	5/24	13/54	0.803
Ao _max_ (mm)	55 (40-89)	53 (45-68)	0.384	52 (41-72)	55 (40-89)	0.171
AsAA	23	24	3.0E-5	12/17	35/32	0.328
BAV	—	—	—	12/17	18/49	0.159
AVR ≥ 2	49	18	0.159	—	—	—

^∗^Chi^2^ test for qualitative variables and Mann-Whitney test for quantitative variables. TAV: tricuspid aortic valve; BAV: bicuspid aortic valve; AVR: aortic valve regurgitation; BMI: body mass index; CAD: coronary artery disease; Ao _max_: maximal diameter of aorta; AsAA: ascending aortic aneurysm; *p*_B_: BAV patients versus TAV patients; *p*_AI_: AVR ≥ 2 patients versus AVR ≤ 1 patients.

**Table 3:** The frequency distribution of *MMP1*, *MMP9*, *COL1A1*, and *COL1A2* variants in the studied patients.

Polymorphism^∗^	Control group (*n* = 61)	AsAA (*n* = 47)	TAD (*n* = 49)	*p*_CvsA_	*p*_CvsT_	*p*_AvsT_
	*n* (%)	*n* (%)	*n* (%)			
rs1799750 *MMP1*						
1G1G	23 (37.7)	14 (29.8)	15 (30.6)				
1G2G	27 (44.3)	24 (51.1)	29 (59.2)	0.683	0.256	0.448	
2G2G	11 (18.0)	9 (19.1)	5 (10.2)				
1G/2G	73/49 (59.8/40.2)	52/42 (55.3/44.7)	59/39 (60.2/39.8)	0.506	0.956	0.494	
rs3918242 *MMP9*						
CC	45 (73.8)	37 (78.7)	37 (75.5)				
CT	13 (21.3)	10 (21.3)	11 (22.5)	0.302	0.724	0.605	
TT	3 (4.9)	0 (0.0)	1 (2.0)				
C/T	103/19 (84.4/15.6)	84/10 (89.4/10.6)	85/13 (86.7/13.3)	0.292	0.630	0.576	
rs1800012 *COL1A1*						
GG	38 (62.3)	31 (66.0)	34 (69.4)				
GT	21 (34.4)	14 (29.8)	13 (26.5)	0.861	0.669	0.936	
TT	2 (3.3)	2 (4.2)	2 (4.1)				
G/T	97/25 (79.5/20.5)	76/18 (80.8/19.2)	81/17 (82.6/17.4)	0.807	0.556	0.747	
rs42524 *COL1A2*						
CC	35 (57.4)	18 (38.3)	25 (51.0)				
CG	22 (36.1)	25 (53.2)	22 (44.9)	0.143	0.596	0.376	
GG	4 (6.6)	4 (8.5)	2 (4.1)				
C/G	92/30 (75.4/24.6)	61/33 (64.9/35.1)	72/26 (73.5/26.5)	0.092	0.743	0.198	

^∗^Chi^2^ test for genotypes and alleles; AsAA: ascending aortic aneurysm; TAD: thoracic aortic dissection; *p*_CvsA_: control subjects versus AAA patients; *p*_CvsT_: control subjects versus TAD patients; *p*_AvsT_: AAA patients versus AAA patients.

**Table 4:** *MMP1*, *MMP9*, *COL1A1*, and *COL1A2* variants in patients with aortopathy in regard to the type of aortic valve.

Polymorphism	TAV group (*n* = 66)	BAV group (*n* = 30)	*p* ^∗^	Compared genotypes or alleles	OR (95% CI) BAV vs. TAV	*p* ^#^
	*n*	*n*				
rs1799750 *MMP1*						
1G1G	21	8	0.581	2G2G+1G2G vs. 1G1G	1.28 (0.49-3.35)	0.810
1G2G	37	16		2G2G vs. 1G2G+1G1G	1.81 (0.57-1.81)	0.356
2G2G	8	6		2G vs. 1G	1.30 (0.71-2.41)	0.432
rs3918242 *MMP9*						
CC	51	23	0.780	TT+CT vs. CC	1.03 (0.37-2.88)	0.948
CT	14	7		TT vs. CT+CC	0 (-)	0.498
TT	1	0		T vs. C	0.96 (0.37-2.47)	0.928
rs1800012 *COL1A1*						
GG	48	17	0.276	TT+GT vs. GG	2.04 (0.83-5.03)	0.158
GT	16	11		TT vs. GT + GG	2.29 (0.31-17.05)	0.586
TT	2	2		T vs. G	1.87 (0.88-3.97)	0.110
rs42524 *COL1A2*						
CC	35	8	0.049	GG+CG vs. CC	3.10 (1.21-7.97)	0.026
CG	28	19		GG vs. CG+CC	2.33 (0.44-12.30)	0.372
GG	3	3		G vs. C	2.06 (1.08-3.92)	0.029

TAV: tricuspid aortic valve; BAV: bicuspid aortic valve. Genotype-phenotype associations are presented as odds ratios (OR) with 95% confidence intervals (95% CI) under dominant, recessive, and additive model of inheritance. ^∗^Chi-squared test. ^#^Fisher's exact test.

**Table 5:** *MMP1*, *MMP9*, *COL1A1*, and *COL1A2* variants in patients with aortopathy in regard to the degree of aortic valve regurgitation.

Polymorphism	AVR ≤ 1 (*n* = 29)	AVR ≥ 2 (*n* = 67)	*p* ^∗^	Compared genotypes or alleles	OR (95% CI) AVR ≥ 2 vs. AVR ≤ 1	*p* ^#^
	*n*	*n*				
rs1799750 *MMP1*						
1G1G	3	26	0.020	2G2G+1G2G vs. 1G1G	0.18 (0.05-0.66)	0.007
1G2G	21	32		2G2G vs. 1G2G+1G1G	0.74 (0.23-2.45)	0.754
2G2G	5	9		2G vs. 1G	0.52 (0.28-0.97)	0.040
rs3918242 *MMP9*						
CC	19	55	0.099	TT+CT vs. CC	0.41 (0.15-1.11)	0.111
CT	9	12		TT vs. CT+CC	0 (-)	0.302
TT	1	0		T vs. C	0.42 (0.17-1.018)	0.056
rs1800012 *COL1A1*						
GG	15	50	0.003	TT+GT vs. GG	0.36 (0.15-0.91)	0.034
GT	10	17		TT vs. GT+GG	0 (-)	0.007
TT	4	0		T vs. G	0.32 (0.15-0.69)	0.004
rs42524 *COL1A2*						
CC	12	31	0.905	GG+CG vs. CC	0.82 (0.34-1.98)	0.823
CG	15	32		GG vs. CG+CC	0.86 (0.15-4.96)	1.000
GG	2	4		G vs. C	0.87 (0.45-1.69)	0.734

AVR: aortic valve regurgitation. Genotype-phenotype associations are presented as odds ratios (OR) with 95% confidence intervals (95% CI) under dominant, recessive, and additive model of inheritance. ^∗^Chi-squared test. ^#^Fisher's exact test.

**Table 6:** Multivariate logistic regression analysis for the prediction of large aortic valve regurgitation (AVR ≥ 2) in patients with aortopathy.

Independent variables	OR (95% CI)	*p* value
*MMP1* (at least one 2G allele)	0.03 (0.01–0.24)	0.001
*MMP9* (number of T alleles)	0.11 (0.03–0.49)	0.003
*COL1A1* (number of T alleles)	0.28 (0.10–0.78)	0.013
AsAA	6.25 (1.31–33.3)	0.019
Maximal diameter of aorta (mm)	1.09 (1.01–1.19)	0.033
BAV	0.26 (0.06–1.12)	0.066
DM	0.08 (0.01–0.83)	0.032

OR: odds ratio; (95% CI): 95% confidence interval; AsAA: ascending aortic aneurysm; BAV: bicuspid aortic valve; DM: diabetes mellitus.

## Data Availability

Data is available on request through the corresponding author.
